# Identification of CD8^+^ T cell epitopes through proteasome cleavage site predictions

**DOI:** 10.1186/s12859-020-03782-1

**Published:** 2020-12-14

**Authors:** Marta Gomez-Perosanz, Alvaro Ras-Carmona, Esther M. Lafuente, Pedro A. Reche

**Affiliations:** Laboratory of Immunomedicine, Department of Immunology, Faculty of Medicine, Complutense University of Madrid, Pza Ramon y Cajal, s/n, 28040 Madrid, Spain

**Keywords:** Proteasome, Immunoproteasome, Prediction, Peptide, CD8^+^ T cell epitope, SARS-CoV-2

## Abstract

**Background:**

We previously introduced PCPS (Proteasome Cleavage Prediction Server), a web-based tool to predict proteasome cleavage sites using *n-grams*. Here, we evaluated the ability of PCPS immunoproteasome cleavage model to discriminate CD8^+^ T cell epitopes.

**Results:**

We first assembled an epitope dataset consisting of 844 unique virus-specific CD8^+^ T cell epitopes and their source proteins. We then analyzed cleavage predictions by PCPS immunoproteasome cleavage model on this dataset and compared them with those provided by a related method implemented by NetChop web server. PCPS was clearly superior to NetChop in term of sensitivity (0.89 vs. 0.79) but somewhat inferior with regard to specificity (0.55 vs. 0.60). Judging by the Mathew’s Correlation Coefficient, PCPS predictions were overall superior to those provided by NetChop (0.46 vs. 0.39). We next analyzed the power of C-terminal cleavage predictions provided by the same PCPS model to discriminate CD8^+^ T cell epitopes, finding that they could be discriminated from random peptides with an accuracy of 0.74. Following these results, we tuned the PCPS web server to predict CD8^+^ T cell epitopes and predicted the entire SARS-CoV-2 epitope space.

**Conclusions:**

We report an improved version of PCPS named iPCPS for predicting proteasome cleavage sites and peptides with CD8^+^ T cell epitope features. iPCPS is available for free public use at https://imed.med.ucm.es/Tools/pcps/.

## Background

Proteasomes are multicatalytic protease complexes that play a central role in cellular protein homeostasis by degrading damaged and misfolded proteins [1–3]. Within the cell, the majority of proteins destined to degradation are marked with ubiquitin and delivered to the proteasome, which cut them into peptide fragments that are then easily degraded by other proteases up to recoverably amino acids [4]. Proteasomes can also degrade proteins through ubiquitin-independent pathways [5] and, in vertebrates, are key components of the class I antigen presentation pathway [6]. Degradation of intracellular proteins by proteasomes produces peptides that can eventually bind to major histocompatibility complex (MHC I) molecules and be presented to CD8^+^ T cells. Moreover, it has been shown that the C-terminus of peptides presented by MHC I molecules results from proteasome cleavage [7].

The essential role of the proteasomes in class I antigen presentation has been elucidated in mammals, where two main forms of the proteasome exist, the constitutive proteasome and the immunoproteasome [8]. Most cells generally express the constitutive or standard proteasome, but under proinflammatory stimuli they switch to express the immunoproteasome [9, 10]. The immunoproteasome is also constitutively expressed by some immune cells and in particular by dendritic cells, professional antigen presenting cells responsible for priming T cells [11]. In the immunoproteasome, the catalytic β1, β2 and β5 subunits of the standard proteasome, are replaced by the subunits β1i (Low-molecular mass protein-2, LMP2), β2i (Multicatalytic endopeptidase complex-like 1, MECL-1) and β5i (Low-molecular mass protein-7, LMP7), respectively [12]. As a result, proteasomes and immunoproteasomes cleave proteins at different sites [13]. In particular, the immunoproteasome does not cut after acidic residues and have a higher cleavage preference after hydrophobic and basic residues. Overall, the proteolytic activity of the immunoproteasome is optimized to provide peptide antigens for presentation by MHC I molecules and most CD8^+^ T cell epitopes result from immunoproteasome cleavage [14].

Given that proteasomes/immunoproteasomes determine the repertoire of CD8^+^ T cell epitopes, researchers have developed different approaches to predict proteasome cleavage sites [15]. Since the C-terminus of the peptides presented by MHC I molecules correspond to the P1 residue of the cleavage site, we and others have produced models to predict proteasome cleavage sites that are trained on datasets consisting of MHC I peptide ligands and their C-terminal flanking regions [16–18]. The task of modeling cleavage sites resemble that of modeling grammatical rules and we specifically used *n-grams* to model and predict immunoproteasome cleavage sites [16]. Moreover, we developed a web-based tool, PCPS (Proteasome Cleavage Prediction Server), implementing these *n-grams* models for free public use at https://imed.med.ucm.es/Tools/pcps/.

Proteasomal cleavage site predictions serve to enhance CD8^+^ T cell epitope discrimination when combined with peptide-MHC I binding predictions [16, 19, 20]. However, here we sought to analyze if cleavage predictions provided by PCPS immunoproteasome models could alone serve to predict CD8^+^ T cell epitopes. Using a dataset of 844 virus-specific CD8^+^ T cell epitopes, we discriminated epitopes from random peptides with an accuracy of 0.74 or more, regardless of the presenting MHC I molecules. Following these results, we have enhanced PCPS to a new version named iPCPS, enabling CD8^+^ T cell epitope prediction and we applied the tool to identify the entire Severe Acute Respiratory Syndrome Coronavirus 2 (SARS-CoV-2) epitome.

## Results and discussion

### Evaluation of cleavage site predictions by PCPS immunoproteasome model

The C-terminus of most peptides recognized by CD8^+^ T cells in the context of MHC I molecules results from cleavage by the immunoproteasome [21]. Prediction of immunoproteasome cleavage sites is thus relevant for T cell epitope vaccine design and we previously reported *n-grams* models for such a task that were implemented in PCPS online tool. In cross-validation, PCPS immunoproteasome *n-gram*s reached an MCC of 0.47. Here, we evaluated cleavage predictions by the default PCPS immunoproteasome model using a larger independent dataset consisting of 257 proteins encompassing 844 9-mer virus-specific CD8^+^ T cell epitopes (see Additional file 1). These CD8^+^ T cell epitopes were obtained from the IEDB database [22] and were reported to be recognized by humans during the course of a viral infection (details in "Methods"). CD8^+^ T cells are primed by peptide antigens processed and presented by dendritic cells which express the immunoproteasome. Therefore, all the selected epitopes ought to be generated by the immunoproteasome [23, 24].

To evaluate cleavage site prediction on this dataset we followed the assumption that cleavage is more likely to occur in the C-terminus of the epitopes than in any other internal cleavage site [25]. Under this assumption, CD8^+^ T cell epitopes with internal cleavage sites with a score above that of the C-terminus were considered false positives (details in "Methods"). We also carried out the same analysis using the immunoproteasome model of NetChop, which is often considered a reference tool for immunoproteasome cleavage site predictions [17, 25]. In both methods, we used a default score of 0.5 as the threshold to define cleavage sites.

According to the results, summarized in Table 1, the specificity of the predictions by PCPS was somewhat lower than that of NetChop (0.55 vs. 0.60, respectively). However, the sensitivity of PCPS predictions was clearly superior to those obtained with NetChop (0.89 *vs.* 0.79, respectively). Moreover, judging by Mathews Correlation Coefficient, PCPS performance was overall better than that of NetChop (0.46 *vs.* 0.39, respectively). It is worth noting that we used the original PCPS *n-gram* models while NetChop models have been retrained in increasingly larger datasets (current version is 3.1), indicating that we already have enough data to capture proteasome cleavage sites.

**Table 1:** Predictive performance of PCPS and NetChop

Server	SE	SP	MCC
PCPS	0.89	0.55	0.46
NetChop	0.79	0.60	0.39

*SE* sensitivity, *SP* specificity, *MCC* Mathews correlation coefficient of the cleavage prediction method as computed by Eqs. 1, 2 and 4 (see "Methods")

### Discrimination of CD8^+^ T cell epitopes by C-terminal cleavage predictions

**Fig. 1:**
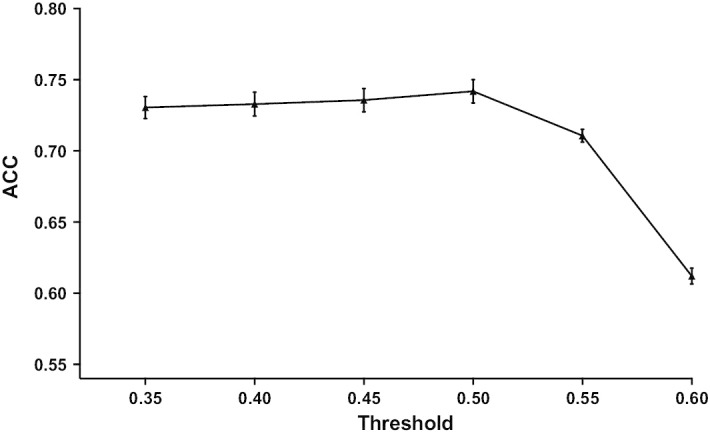
Discrimination of CD8^+^ T cell epitopes using PCPS immunoproteasome cleavage predictions. Graph depicts the accuracy (ACC) of PCPS immunoproteasome model to distinguishing CD8^+^ T cell epitopes by their C-terminus at different thresholds (0.35–0.6). The analysis to compute ACC was repeated 5 times with different 9-mer peptides selected randomly from the same source proteins than the epitopes. Average values of ACC are represented with standard deviations as errors bars

CD8^+^ T cells only recognize peptides presented by MHC I molecules and prediction of peptide-MHC I binding is therefore the main basis for anticipating CD8^+^ T cell epitopes [26]. Proteasomal cleavage is typically used in combination with MHC I-binding predictions to enhance CD8^+^ T cell epitope discrimination [15]. In particular, it has been shown that combining PCPS cleavage predictions with MHC I binding predictions reduces the number of false positives around a 70% [16]. Here we analyzed if cleavage predictions alone could serve to predict CD8^+^ T cell epitopes. To that end, we tested the ability of the PCPS immunoproteasome model to distinguish our set of 844 CD8^+^ T cell epitopes by cleavage at their C-terminus in their source proteins with regard to random peptides generated from the same proteins. The results obtained at different cleavage site score thresholds are summarized in Fig. 1. The epitopes were predicted with an accuracy of over 0.70 for thresholds from 0.35 to 0.55, reaching a top accuracy of 0.74 ± 0.01 at the 0.5 threshold. The sensitivity and specificity of the predictions at this same threshold were 0.89 ± 0.01 and 0.60 ± 0.01, respectively. Note that these results were obtained without considering the restriction elements of the CD8^+^ T cell epitopes.

**Fig. 2:**
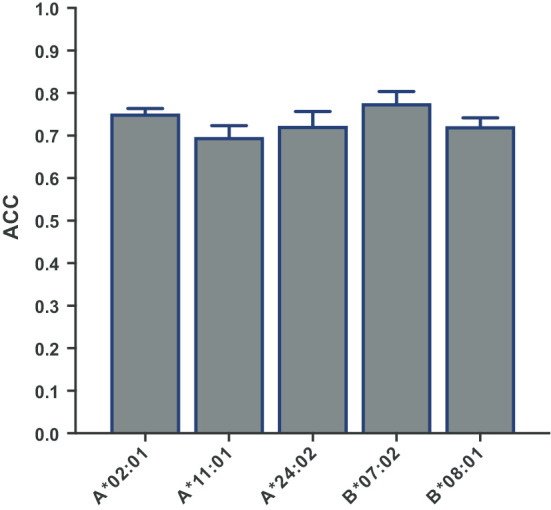
Discrimination of CD8^+^ T cell epitopes by immunoproteasome cleavage predictions considering HLA I restriction. Figure shows the accuracy (ACC) of C-terminus immunoproteasome cleavage predictions discriminating virus-specific CD8^+^ T cell epitopes restricted by specific HLA I molecules (columns) from 9-mer peptides, selected randomly from the same source proteins than the epitopes. The analysis was carried out using PCPS immunoproteasome model at a threshold of 0.5 and the represented ACC corresponds to means ± SD obtained after repeating the analysis five times with different random 9-mer peptides

We also analyzed the epitope prediction results obtained with the best threshold (0.5) with regard to the human leukocyte antigen class I (HLA I) molecules known to restrict the CD8^+^ T cell responses (see Additional file 1). Thus, we selected the CD8^+^ T cell epitopes restricted by HLA-A*02:01, HLA-A*11:01, HLA-A*24:02, HLA-B*07:02 and HLA-B*08:01 and computed the performance separately. HLA I molecules are highly polymorphic [27] and the selected HLA I molecules are frequently expressed in the world population. As shown in Fig. 2, the selected CD8^+^ T cell epitopes could be predicted with an accuracy that ranged from 0.70 ± 0.02 for those restricted by HLA-A*11:01 and 0.78 ± 0.03 for those restricted by HLA-B*07:02.

In sum, our results clearly show that the prediction of C-terminal cleavage sites in peptides by PCPS immunoproteasome model alone can serve to predict CD8^+^ T cell epitopes covering all HLA I molecules, which is of particular relevance when no peptides are available for developing HLA I-binding models. Following these results, we have enhanced PCPS to enable CD8^+^ T cell epitope predictions. In the next section we illustrate the usage of the improved version of PCPS, which we renamed as iPCPS.

### iPCPS usage and SARS-CoV-2 epitome analysis

**Fig. 3:**
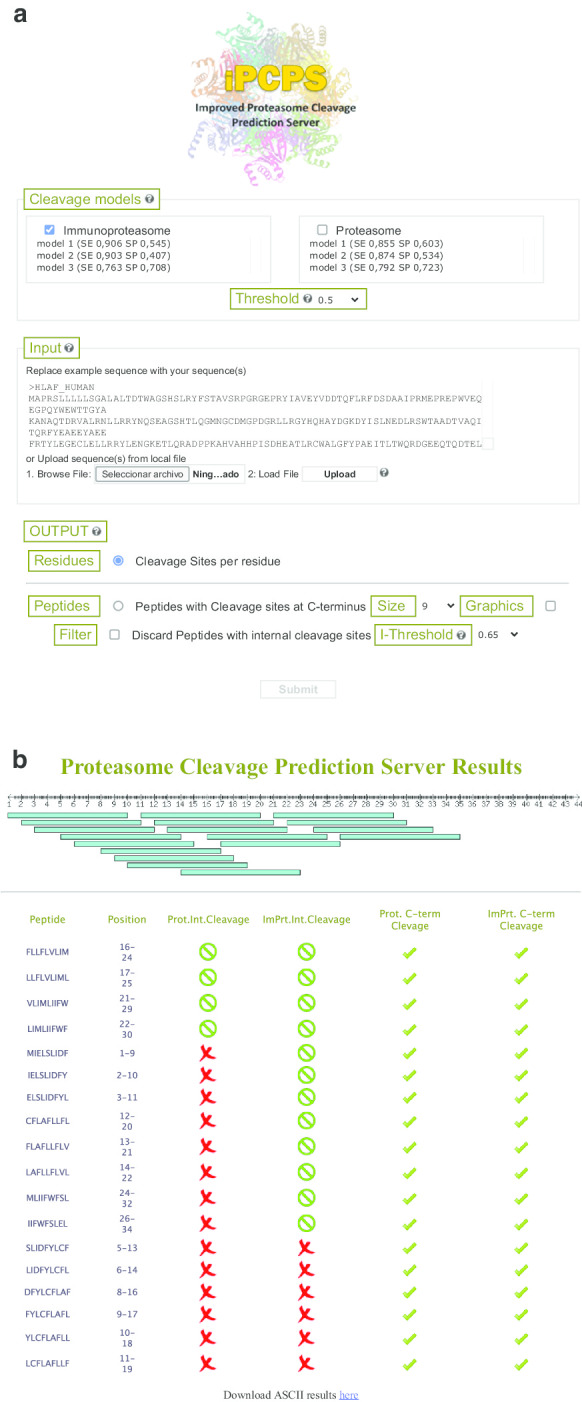
Improved Proteasome Cleavage Prediction Server (iPCPS). **a** Web interface of iPCPS. The interface of iPCPS is divided in three sections for intuitive use. In the first section (models), users select the immunoproteasome and/or proteasome model and the threshold. In input, users paste or upload their sequence/s in FASTA format and in output select to get cleavage sites per residue or peptides with C-terminus generated by the proteasome. Users can set the size of the peptide, discard those with internal cleavage sites and obtain a graphical display. **b** iPCPS output. Figure shows a representative iPCPS peptide output obtained selecting the proteasome and immunoproteasome model and graphics display. Ticks indicate that peptides have a C-terminus generated by the proteasomes and green zero symbols are for peptides without internal cleavage sites for the selected threshold. Peptides with red cross symbols do have internal cleavage sites

PCPS was initially developed to predict proteasome cleavage sites in amino acid sequences using *n-grams.* This option is still available in iPCPS (Fig. 3a), but the new version, can also return all the peptides (length selected by users) with a C-terminus compatible with immunoproteasome cleavage (Fig. 3b). We recommend using the default length of 9 residues, as this is the most common size of peptides presented by MHC I molecules. It is known that immunoproteasomes can also destroy potential CD8^+^ T cell epitopes [28]. Therefore, we implemented the possibility of discarding from the output those peptides with internal cleavage sites. This feature can also lead to the loss of true CD8^+^ T cell epitopes. Users can set both the cleavage site score thresholds for C-terminal and internal cleavage sites predictions. By default, the threshold for internal cleavage sites is set at 0.65, higher than that for C-terminal cleavage sites to minimize the loss of *bona fide* CD8^+^ T cell epitopes.

iPCPS also includes *n-grams* models for the constitutive proteasome which users can select instead of or in combination with immunoproteasome models. Constitutive proteasomes also have a role in the generation of the repertoire of peptides that can be presented by MHC I molecules, particularly in non-immune cells targeted by already primed CD8^+^ T cells [29]. Proteasome models available on iPCPS were trained on self- peptides eluted from human MHC I ligands instead of on CD8^+^ T cell epitopes [16]. When users combine immunoproteasome and proteasome models, iPCPS will return the repertory of peptides that have a C-terminus compatible with cleavage by both, the immunoproteasome and the proteasome. Protective CD8^+^ T cell epitopes are thought to be generated by both types of proteasome [30]. If user selects the option “Discard peptides with internal cleavage sites” with the combination of immunoproteasome and proteasome models, iPCPS will return those peptides without internal cleavage sites by both models.

**Fig. 4:**
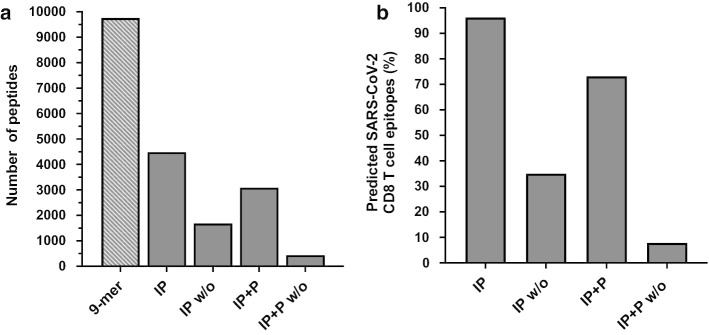
Predicted SARS-CoV-2-specific CD8^+^ T cell epitopes using iPCPS. **a** The figure represents the number of predicted CD8^+^ T cell epitopes in SARS-CoV-2 proteome using iPCPS with different settings indicated in the abscissa: 9-mer, total 9-mer peptides in the SARS-CoV-2 proteome; IP, immunoproteasome model; IP w/o, immunoproteasome model discarding peptides with internal cleavage sites; IP + P, immunoproteasome and proteasome models; IP + P w/o, immunoproteasome and proteasome models discarding peptides with internal cleavage sites. **b** Percentage of SARS-CoV-2 CD8^+^ T cell epitopes predicted by peptide-HLA I binding predictions reported by Grifoni et al. [31] included in those anticipated by iPCPS with settings indicated in panel A

To illustrate the full potential of iPCPS, we predicted CD8^+^ T cell epitopes in SARS-CoV-2 (ACN: NC_045512.2) with different settings and compared them with those predicted by Grifoni et al. [31] for 12 common HLA I molecules (Fig. 4). We only focused on peptides with a size of 9 residues. The complete epitope space of SARS-CoV-2 encompassing all 9-mer peptides is 9757. Using the iPCPS immunoproteasome model with default settings, we identified 4486 peptides with a C-terminus compatible with immunoproteasomal processing. This set of peptides contain epitopes restricted by all HLA I molecules and actually includes 96% of the epitopes predicted by Grifoni et al. [31]. Note that there are hundreds of HLA I molecules which actually exhibit distinct peptide binding specificities [27]. If we discard those peptides with internal cleavage sites, the set of predicted CD8^+^ T cell epitopes drop down to 1682 (Fig. 4a), and includes only 35% of the predicted CD8^+^ T cell epitopes after their MHC I binding (Fig. 4b). We get 3091 peptides when combining immunoproteasome and proteasome cleavage models (Fig. 4a), encompassing 73% of the predicted CD8^+^ T cell epitopes (Fig. 4b). If we again discard those peptides with internal cleavage sites we obtain 437 CD8^+^ T cell epitopes (Fig. 4a), including only 8% of the CD8^+^ T cell epitopes resulting from HLA I binding predictions (Fig. 4b). Despite we are analyzing iPCPS results on predicted CD8^+^ T cell epitopes and not actual epitopes, our comparison indicates that, overall, the best way for predicting CD8^+^ T cell epitopes in iPCPS is using immunoproteasome models alone or in combination with proteasome models. Discarding peptides with internal cleavage sites can lead to a great loss of *bona fide* CD8^+^ T cell epitopes, likely reflecting that proteasomes are quite unspecific, as their main role is the complete degradation of intracellular proteins rather than the generation of CD8^+^ T cell epitopes. Nonetheless, the option of discarding peptides with internal cleavage sites may be useful with large proteomes as it narrows down the number of potential epitopes for experimental scrutiny. CD8^+^ T cell epitopes predicted with iPCPS using different settings are provided in Additional file 2.

## Conclusion

**Fig. 5:**
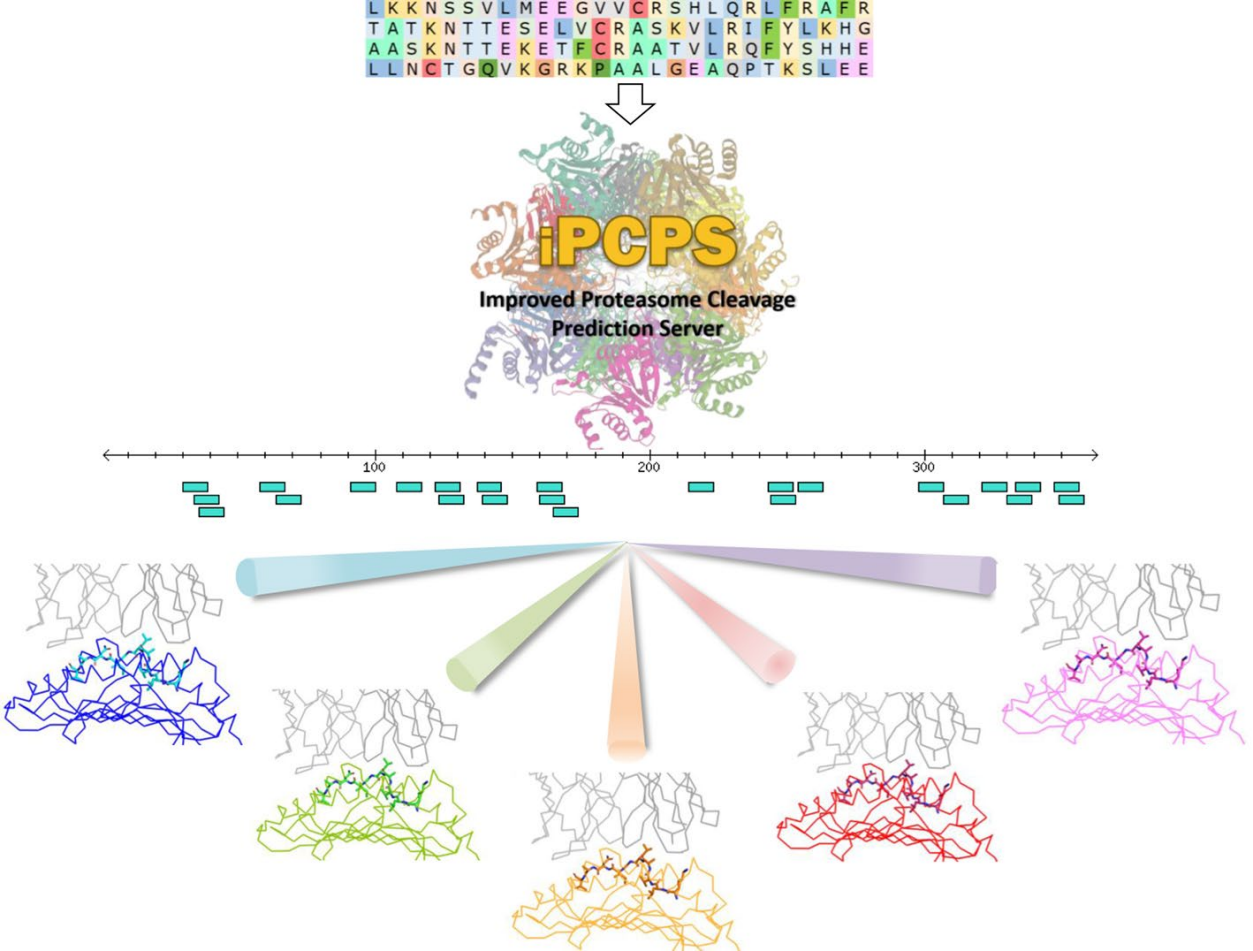
iPCPS graphic work. The figure depicts the suitability of iPCPS to predict potential CD8^+^ T cell epitopes covering all HLA I molecules

We describe an improved version of PCPS named iPCPS for predicting proteasome cleavage sites and peptides with CD8^+^ T cell epitope features, as depicted in Fig. 5. To our knowledge, iPCPS is the only tool for predicting proteasome cleavage sites with such capability. iPCPS is available for free public use at https://imed.med.ucm.es/Tools/pcps/.

## Methods

### Epitopes and sequences

We obtained virus-specific CD8^+^ T cell epitopes from the Immune Epitope Database (IEDB) [22]. We limited our search to epitope peptides from virus that gave positive T cell responses in the course of a natural infection in humans and were restricted by MHC I molecules. Subsequently, we selected those epitopes with a length of 9 residues, the optimal for MHC I binding, and those whose entire sequence matched exactly with that of relevant source proteins. Amino acid sequences in FASTA format of epitope source proteins were obtained from UNIPROT (https://www.uniprot.org/) after the accession numbers (ACN) provided by IEDB.

### Prediction of proteasome cleavage sites

We predicted proteasome cleavage sites using PCPS [16]. PCPS implements *n-grams* models that were trained on fragments obtained upon peptides eluded from human MHC I molecules (proteasome models) and upon naturally restricted CD8^+^ T cell epitopes (immunoproteasome models). In this work, we selected the default immunoproteasome model, which was specifically trained on 8 residue peptide fragments, each including a cleavage site (P1 and P1′ residues). Peptide fragments included the 4 last residues of CD8^+^ T cell epitopes followed by 4 proximal residues that flank the C-terminus of the epitopes in the relevant source proteins. We compared PCPS cleavage site predictions with those provided by NetChop (https://www.cbs.dtu.dk/services/NetChop/) [17]. In NetChop, we selected the default C-term 3.0 model, equivalent to the PCPS immunoproteasome model. NetChop C-term 3.0 was trained on peptide fragments generated upon MHC I peptide ligands and provides the most accurate cleavage predictions [17].

### Evaluation of proteasome cleavage site predictions

We evaluated the performance of PCPS and NetChop immunoproteasome models on sequences containing CD8^+^ T cell epitopes following the approach described elsewhere [16, 25] and considering a default cleavage threshold (*Th)* of 0.5. This approach assumes that cleavage after the epitope C-terminus has to be favored with regard to other cleavage sites within the epitope (internal cleavage site). Briefly, by evaluating cleavage scores (*Cs*) on each epitope residue, cleavage sites defined by epitopes were classified as follows:

- True positive (TP): The C-terminus *Cs* is ≥ *Th*.
- False Positive (FP): At least one internal *Cs* is ≥ *Th* and ≥ than that of the C-terminus.
- True Negative (TN): All internal *Cs* are < *Th* or < than that of the C-terminus.
- False Negative (FN): The C-terminus *Cs* is < *Th.*

The performance of the predictions was then measured in terms of Sensitivity (SE), Specificity (SP), Accuracy (ACC) and Matthews Correlation Coefficient (MCC) using Eqs. 1, 2, 3 and 4.

1 $$SE = \frac{TP}{{TP + FN}}$$

2 $$SP = \frac{TN}{{TN + FP}}$$

3 $$ACC = \frac{TP + TN}{{TP + FP + TN + FN}}$$

4 $$MCC = \frac{{\left( {TP*TN} \right) - \left( {FN*FP} \right)}}{{\sqrt {\left( {TN + FN} \right)\left( {TP + FN} \right)\left( {TN + FP} \right)\left( {TP + FP} \right)} }}$$

### Evaluation of CD8^+^ T cell epitope prediction

The capacity of C-terminal cleavage predictions provided by PCPS immunoproteasome model to anticipate CD8^+^ T cell epitopes was analyzed by determining their ability to discriminate viral CD8^+^ T cell epitopes from random 9-mer peptides. Random peptides were considered as non-epitopes and were selected randomly from the same protein sources than the CD8^+^ T cell epitopes at a 1:1 ratio. Cleavage sites predictions were carried over the protein sources containing both epitopes and randomly selected peptides. TPs, TNs, FPs and FNs were obtained at different cleavage thresholds (0.35, 0.40, 0.45, 0.5, 0.55 and 0.60) by examining the cleavage scores after the C-terminal residue of both CD8^+^ T cell epitopes (positive instances) and random peptides (negative instances). CD8^+^ T cell epitopes with a cleavage score above threshold were TPs and with cleavage scores below threshold were FNs. Conversely, random 9-mer peptides with a cleavage score below threshold were TNs and those with cleavage scores above threshold were FPs. The performance of the predictions was subsequently assessed by computing SE, SP and ACC, using Eqs. 1, 2 and 3. These analyses were repeated five times, each time selecting different random peptides, obtaining average performance values with standard deviations.

## Supplementary information

**Additional file 1.** Dataset with virus-specific CD8^+^ T cell epitopes.

**Additional file 2.** SARS-CoV-2-specific CD8^+^ T cell epitopes predicted using iPCPS with different settings.

## Data Availability

Authors confirm that all relevant data are included in the article and/or its supplementary information files.

## Abbreviations

HLA I: Human leukocyte antigen class I
LMP2: Low-molecular mass protein-2
LMP7: Low-molecular mass protein-7
MECL-1: Multicatalytic endopeptidase complex-like 1
SARS-CoV-2: Severe acute respiratory syndrome coronavirus 2
